# FANCC localizes with UNC5A at neurite outgrowth and promotes neuritogenesis

**DOI:** 10.1186/s13104-018-3763-1

**Published:** 2018-09-12

**Authors:** FengFei Huang, Manel Ben Aissa, Georges Lévesque, Madeleine Carreau

**Affiliations:** 1 Centre Hospitalier Universitaire de Québec-Université Laval, CHUL, 2705 Boul. Laurier, RC-9800, Quebec, QC G1V 4G2 Canada; 2 Department of Psychiatry and Neurosciences, Université Laval, Quebec, QC Canada; 3 Department of Pediatrics, Université Laval, Quebec, QC Canada; 4 Present Address: Medicinal Chemistry and Pharmacognosy, College of Pharmacy, Chicago, IL USA

**Keywords:** Uncoordinated-5A, Neurite outgrowth, Neuritogenesis, Fanconi anemia, FANCC

## Abstract

**Objective:**

The Uncoordinated 5A (UNC5A) protein is part of a family of receptors that play roles in axonal pathfinding and cell migration. We previously showed that the Fanconi anemia C protein (FANCC) interacts with UNC5A and delays UNC5A-mediated apoptosis. FANCC is a predominantly cytoplasmic protein that has multiple functions including DNA damage signaling, oxygen radical metabolism, signal transduction, transcriptional regulation and apoptosis. Given the direct interaction between FANCC and UNC5A and that FANCC interferes with UNC5A-mediated apoptosis, we explored the possibility that FANCC might play a role in axonal-like growth processes.

**Results:**

Here we show that FANCC and UNC5A are localized to regions of neurite outgrowth during neuronal cell differentiation. We also show that absence of FANCC is required for neurite outgrowth. In addition, FANCC seems required for UNC5A expression. Results from this study combined with our previous report suggest that FANCC plays a role in tissue development through the regulation of UNC5A-mediated functions.

## Introduction

The Uncoordinated 5A (UNC5A) protein belongs to the UNC5 human transmembrane receptor family, which includes four homologs UNC5A, UNC5B, UNC5C and UNC5D. UNC5 proteins promote repulsive signals during neural development and differentiation [1–4]. In addition, UNC5 proteins have been proposed to function as ‘dependence receptors’, triggering apoptosis in the absence of the ligand Netrin-1 and sending survival signals when bound to the ligand [5]. UNC5A has also been shown to promote apoptosis independently of Netrin-1, indicating the possibility of other functional ligands for this receptor [4, 6]. In a previous report, we showed that the Fanconi anemia C protein, FANCC, interacts directly with UNC5A via its cytoplasmic death domain. FANCC interaction with UNC5A was also shown to delay UNC5A-mediated apoptosis [7]. FANCC is one of many Fanconi anemia (FA) proteins that act in signaling events following cellular stress including DNA damage and oxidative stress. Fanconi anemia (FA) is a genetic disease associated with defective hematopoiesis, cancer proneness and developmental deficiencies [8, 9]. Although the primary role of FA proteins is associated with hematopoiesis, the work of Sii-Felice et al. has established that FA proteins are required for the development and survival of neural progenitor cells [10]. In addition, gene expression studies have shown that both *FancA* and *FancC* are highly expressed in the developing brain, specifically in the intermediate zone, which contains migrating neurons [11–13]. Furthermore, FA proteins were shown to be upregulated following ethanol-induced brain injury [14]. Given that UNC5A plays a role in axonal pathfinding mechanisms, neuronal differentiation and survival [15], and that FANCC interacts with UNC5A [7], we hypothesized that FANCC may be involved in neuronal differentiation. Consequently, objectives of this study were to explore whether FANCC is required for neurite outgrowth processes.

## Main text

### Methods

#### Plasmids and DNA constructs

All plasmids used have been described previously in [7]. These include HA-tag UNC5A intracellular domain (pCMVzeoUNC5A^ICD^), full-length FANCC (pREP4-FANCC), FANCC N-terminus (pEGFPFANCC^1–306^), FANCC C-terminus (pEGFPFANCC^307–558^) and Myc-tag FANCE (pCDNA3-FANCE). Other vectors included lentiviral vectors coding for shRNA against FANCC (TRCN0000083368 (sh-C1), TRCN0000083369 and TRCN0000083370) or against UNC5A (V2LHS-16512, V2LHS-16513, V2LHS-304038, V2LHS-304039, V2LHS-304040; ThermoFisher Scientific, Mississauga, ON).

#### Antibodies

The antibodies used in this study were as follows: anti-FANCC (Novus Biologicals, NBP1-03280 or 8F3, MABC524, EMD Millipore); anti-FANCE (Novus Biologicals, NBP1-21365); anti-UNC5A (Sigma-Aldrich); anti-HA (12CA5, #11583816001, Roche Diagnostics, Indianapolis, IN); anti-GFP (clone B2; Santa Cruz Biotechnologies; SC-9996); anti-GAPDH (clone1D4, NB300-221, Novus Biologicals); anti-cMyc (Santa Cruz Biotechnologies clone 9E10, SC-40); anti-Tubulin, (clone DM1A, #T6199, Sigma-Aldrich); goat anti-mouse IgG-HRP or goat anti-rabbit IgG-HRP (SantaCruz Biotechnologies, SC-2064 or SC-2004); Donkey anti-rabbit-Alexafluor 488 (A21206), -Alexafluor 555 (A31572) or -Alexafluor 680 (A10043) and Goat anti-mouse-Alexafluor 488 (A28175), -Alexafluor 555 (A32727) or -Alexafluor 680 (A21057; ThermoFisher Scientific). F-actin was labeled with Alexafluor 555 phalloidin (ThermoFisher Scientific; A34055).

#### Cells, cell culture and transfection

HEK293T cells (ATCC, Cedarlane Laboratories) and mouse embryonic fibroblasts obtained from *FancC*^−/−^ and wildtype mice were grown at 37 °C in 5% CO2 in DMEM medium supplemented with 10% FCS. SH-SY5Y cells (ATCC, CRL-2266) were grown in a mixture of DMEM and Ham’s F12 Nutrient Mixture (1:1) with 10% FCS at 37 °C, 5% CO2, followed by transfection using calcium-phosphate or lipofectamine 2000 (ThermoFisher Scientific). For differentiation assays, SH-SY5Y cells were treated with retinoic acid (10 μM; Sigma-Aldrich) for 48 h or recombinant human Netrin-1 (500 ng/ml, R&D systems, #6419-N1) for 4 h prior to immunofluorescence staining. For UNC5A expression and stability experiments, HEK293T cells were transfected with increasing amounts of FANCC, FANCE or UNC5A^ICD^ as indicated in the figure and compared to cells expressing equimolar amounts of each coding vector.

#### Animals

*FancC* knockout mice (*FancC*^−/−^) used in this study have been described previously [16]. *FancC*^−/−^ were maintained into C57Bl/6J background and housed in a SPF Elite facility without any pathogens. All mice had access to mouse chow and water ad libitum. Five to six months-old mice including wild-type littermates used as controls were included in the study. Mice were euthanized according to procedures approved by the Animal Care Committee of Laval University under the guidelines of the Canadian Council on Animal Care in science.

#### Western blotting analysis and RT-qPCR

Mouse tissue extracts and whole cell lysates were subjected to immunoblot. Total cell lysates were prepared in SDS loading buffer (50 mM Tris–HCL, 2% 2-mercaptoethanol, 2% sodium dodecyl sulfate), sonicated and/or boiled, subjected to electrophoresis on a 10% or 12% SDS-polyacrylamide gel, electrotransferred onto a PVDF membrane and probed with antibodies. For RT-qPCR, total RNA was isolated using the RNeasy Mini Kit RNA purification system according to the manufacturer’s instructions (Qiagen) followed by reverse transcription with random hexamer primers using the SuperScript™II protocol as recommended by the manufacturer (ThermoFisher Scientific). Quantitative PCR was performed with 100 nm each of the forward and reverse *Unc5A, Sdha* (succinate dehydrogenase) or *Tbp* (TATA box binding protein) primers using the SYBR Green DNA binding dye and ABI Prism 7000 Sequence Detection System (ThermoFisher Scientific). Dissociation curve profile of each amplicon and product sizes were verified by agarose 2% gel fractionation. The *Unc5A* gene expression profile was normalized to that of *Sdha* and *Tbp*.

#### Immunofluorescence procedure

SH-SY5Y cells were grown on glass coverslips (12-mm diameter) for 24 h under the appropriate culture conditions prior to fixing with 4% paraformaldehyde in PBS for 20 min at room temperature. Cells were permeabilized for 15 min at room temperature with 0.3% Triton X-100 in PBS and incubated with primary antibodies followed by secondary antibodies in PBS with 10% horse serum at the appropriate dilutions as described in the figure legend. The cells were washed 3 times with PBS, and nuclei were labeled with DAPI prior to mounting. Images were acquired using a Nikon E800 fluorescent microscope equipped with a C1 confocal system (Nikon Canada) at 100× magnification.

#### Statistical analyses

Statistical analyses were performed using paired and unpaired two-tailed Student’s t-tests with the GraphPad Prism software (version 5.0b; GraphPad Software Inc., San Diego, CA).

### Results

#### FANCC and UNC5A are required for neurite outgrowth

**Fig. 1:**
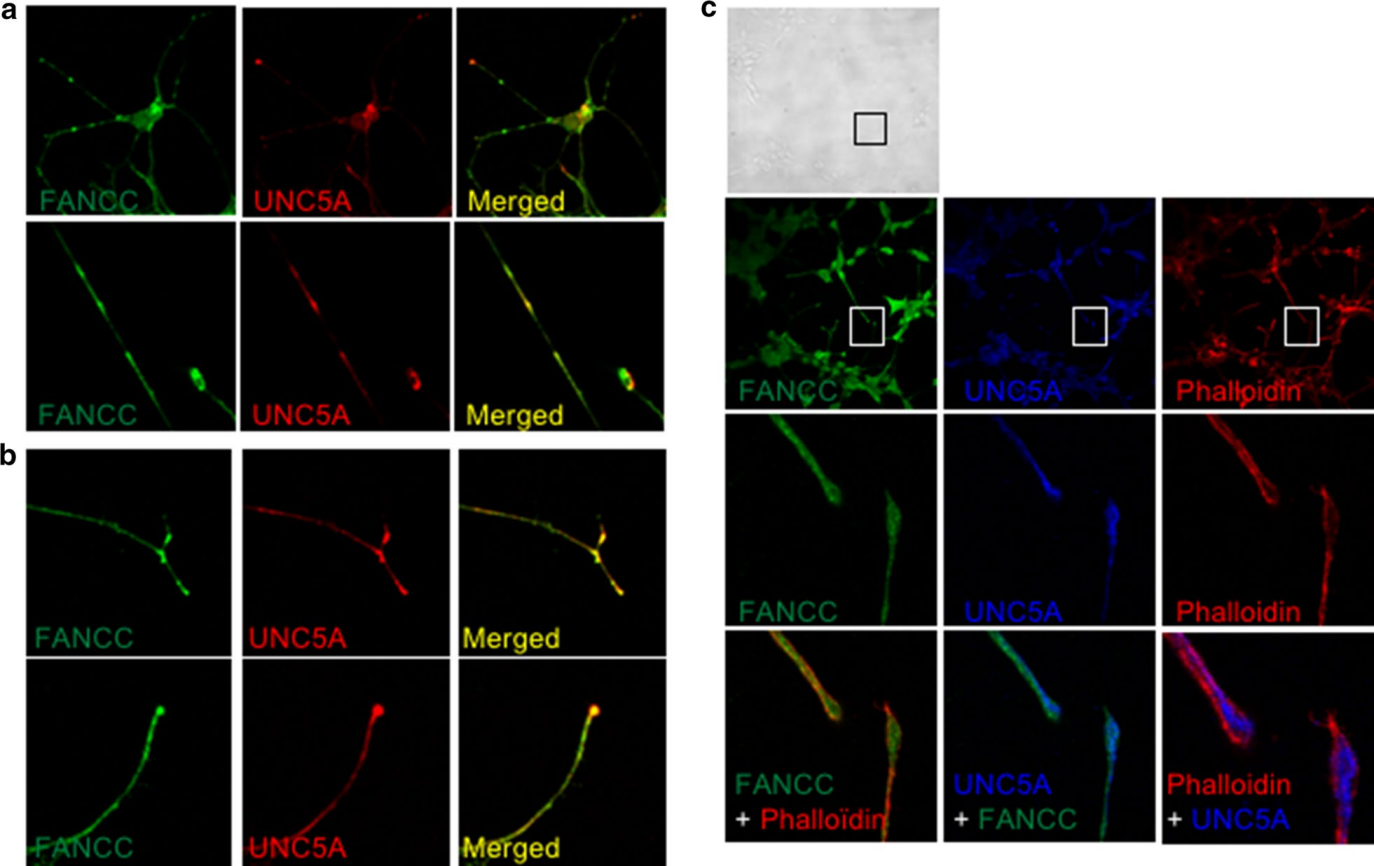
FANCC and UNC5A co-localize to neurite outgrowth. **a**, **b** Representative microscopic images of SH-SY5Y cells incubated with RA (10 μM) for 6 days prior to analysis. Differentiated SH-SY5Y cells were labeled with antibodies against FANCC (green) and UNC5A (red). **c** SH-SY5Y cells were incubated with recombinant Netrin-1 (500 ng/ml) prior to analysis. Labeled cells were visualized by confocal fluorescence microscopy at ×60 and ×100 magnification using a Nikon E800 microscope equipped with a C1 confocal system

To determine whether FANCC localizes with UNC5A to distal projection of neuronal like cells within growth cones and axonal compartments, we used SH-SY5Y neuroblastoma-derived cell lines that we treated with retinoic acid (RA) in order to induce cellular differentiation and neurite-like formation. As expected and consistent with previous reports [17, 18], upon treatment with RA, SH-SY5Y cells showed morphological changes and neurite outgrowth characteristic of neuronal differentiated cells (Fig. 1). Immunofluorescence labeling of differentiated cells shows that both FANCC and UNC5A localized to neurite-like outgrowth structures (Fig. 1a). Importantly, the addition of the UNC5 ligand Netrin-1 during the differentiation process resulted in strong co-labeling of UNC5A with FANCC at the ends of neurite outgrowth (Fig. 1b). This finding was further validated by the labeling of F-actin filaments with a phalloidin conjugate, which confirmed neurite branching and outgrowth in differentiated cells (Fig. 1c). In addition, confocal microscopic analysis confirmed the strong co-labeling of FANCC with UNC5A at regions of growth cones (Fig. 1c).

**Fig. 2:**
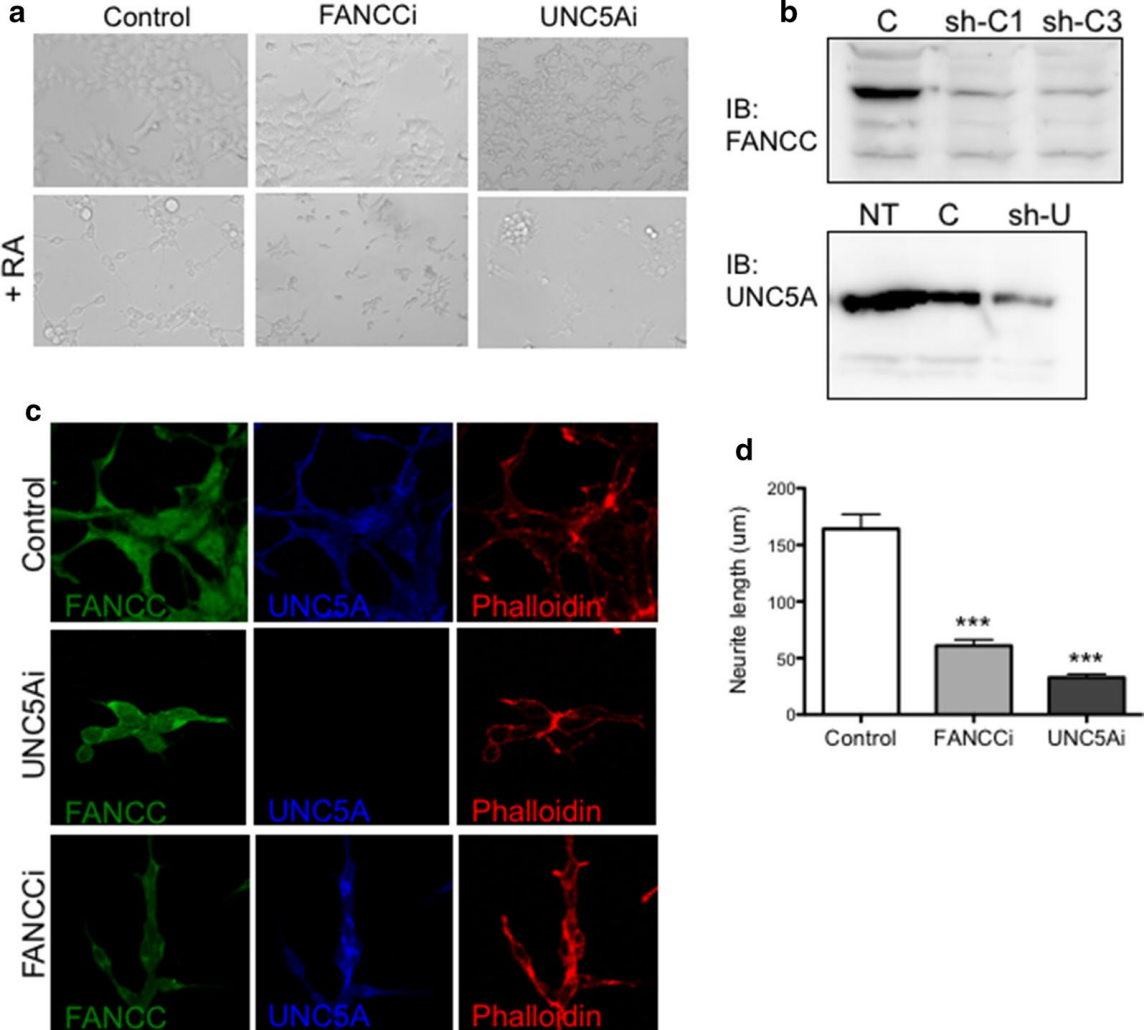
FANCC and UNC5A are required for neurite outgrowth. **a** Representative microscopic images of SH-SY5Y cells stably transduced with shRNA against *FANCC* (FANCCi), *UNC5A* (UNC5Ai), or control noncoding scrambled shRNA (Control) vectors (upper panels) and following differentiation with retinoic acid (+RA at 10μM; lower panels). Cells were visualized at ×40 magnification. **b** Western blots showing the depletion of FANCC using either one shRNA (sh-C1) or a mixture of 3 (sh-C3) against *FANCC* (upper blot) and a mixture of 5 shRNA against *UNC5A* (sh-U; lower blot) in SH-SY5Y cells. **c** Control cells transfected with empty vectors; NT: untransfected cells. **c** *UNC5A*- and *FANCC*-depleted SH-SY5Y cells (UNC5Ai and FANCCi, respectively) induced to differentiate with RA (10μM) were labeled with anti-UNC5A (blue) and anti-FANCC (green) antibodies and phalloidin conjugates (red). The labeled cells were visualized by confocal fluorescence microscopy at ×100 magnification using a Nikon E800 microscope equipped with a C1 confocal system. **d** Estimated neurite length measured estimated Control (n = 15) FANCCi (n = 21) and UNC5Ai (n = 11) ***p > 0.0001

Next, to determine whether FANCC is necessary and required for neurite outgrowth, in the same manner as UNC5A, SH-SY5Y cells were depleted of either FANCC or UNC5A prior to differentiation. Knockdown of UNC5A or FANCC in SH-SY5Y cells resulted in a drastic reduction of differentiated cells and neurite outgrowth upon RA treatment (Fig. 2a). These results are consistent with our previous report showing that UNC5A and FANCC-depleted cells showed reduced cell growth and increased cell death [7]. As shown in Fig. 2b, western blotting experiments confirmed knockdown of FANCC and UNC5A in cells. In addition, labeling of F-actin filaments with phalloidin conjugate confirmed the reduced branching and outgrowth of neurites in cells depleted of either FANCC or UNC5A (Fig. 2c) consistent with a significant reduction in neurite length (Fig. 2d). These results suggest that FANCC and UNC5A are required for neuronal differentiation mechanisms.

#### Unc5A expression is reduced in *FancC*^−/−^ brains

**Fig. 3:**
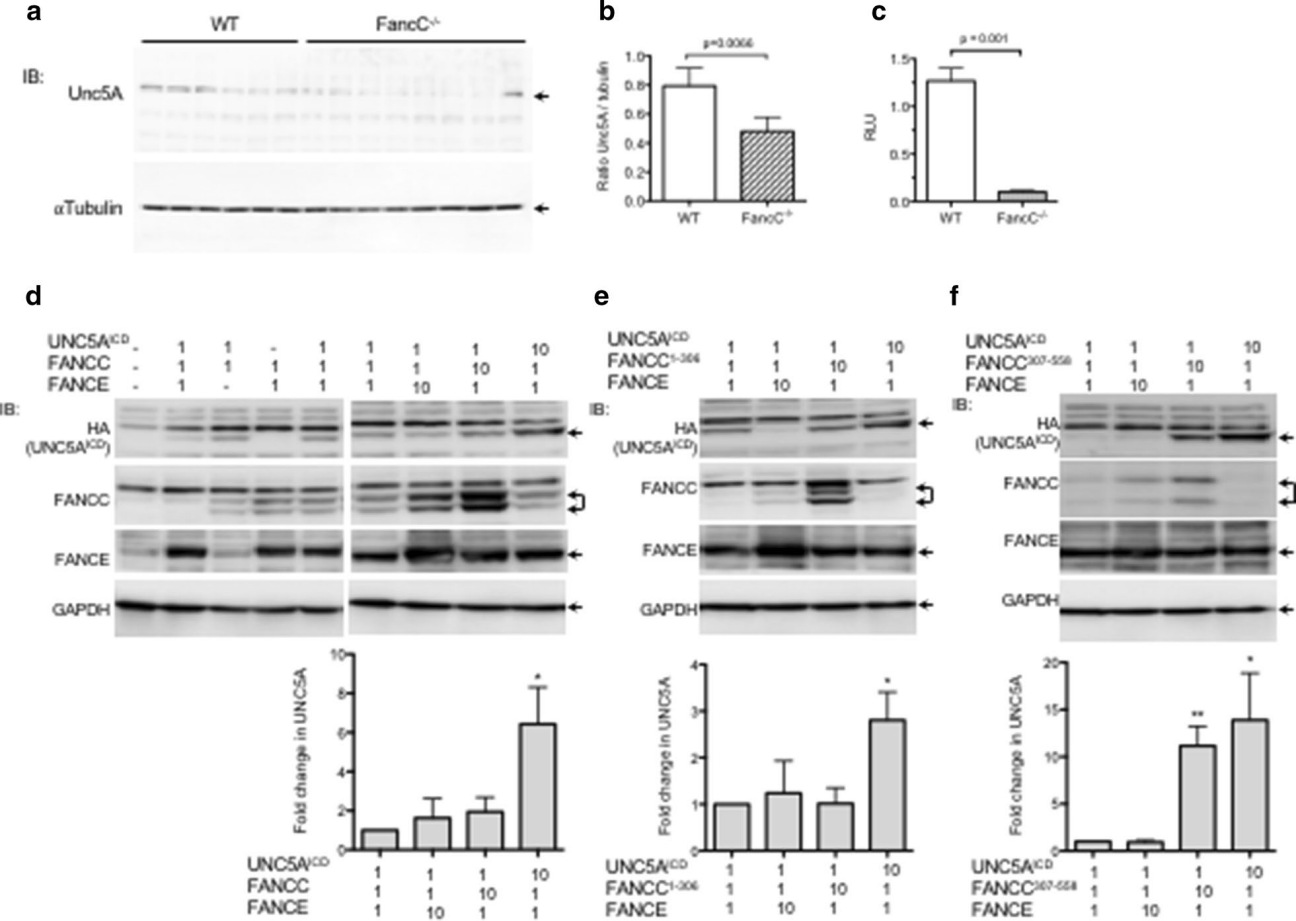
Reduced expression of UNC5A in *FancC*−/− cells. **a** Reduced expression of Unc5A in brain cortex of *FancC*^−/−^ mice compared to wild-type littermates. Western blotting was performed with the indicated antibodies. Each lane represents a different animal (WT: n = 6; *FancC*^−/−^: n = 8). **b** Bar graph represents ratio of Unc5A normalized to tubulin from WT (n = 6) and *FancC*^−/−^ mice (n = 8). **c** Bar graph representation of *UNC5A* expression normalized to *Sdha* and *Tbp* from wild-type (WT) and *FancC*^−/−^-derived fibroblasts (n = 3). RLU: Relative light units. **d**–**f** HEK293T cells were transfected with UNC5A^ICD^, FANCE and FANCC constructs expressing full-length FANCC (in **d**), FANCC^1–306^ (in **e**) or FANCC^307–558^ (in **f**). Constructs were transfected at a molecular ratio of 1:1:1 or with 10 times the molar amount of FANCE, FANCC or UNC5^AICD^ as indicated in the figure. The total amount of transfected plasmid was equalized for all strategies with control empty vectors. Representative immunoblots performed with anti-HA (UNC5A^ICD^), anti-FANCC, anti-FANCE or anti-GAPDH antibodies are shown. Arrows indicate appropriate protein bands. Bar graphs represent the mean fold change ± SEM of UNC5A protein levels normalized to GAPDH compared to 1:1:1 transfection controls in at least 4 separate experiments. *p < 0.05; **p < 0.005

Given that *FancC*^−/−^ mice present decreased neuronal production in developing cortex and adult brain [10], we evaluated Unc5A protein levels in brain cortex of *FancC*^−/−^ mice compared to wild-type littermates. Results show that Unc5A is significantly reduced in the cerebral cortex of *FancC*^−/−^ mice compared to wild-type littermates (Fig. 3a, b). Similarly, we observed reduced *Unc5A* gene expression in *FancC*^−/−^ derived fibroblast cells compared to wild-type cells (Fig. 3c). These results suggest that FANCC may regulate UNC5A expression and/or stability. In line with this postulate, we have previously shown that UNC5A expression levels increased when co-expressed with FANCC [7]. Therefore, to investigate whether UNC5A levels are modulated by FANCC, we co-expressed UNC5A intracellular domain (UNC5A^ICD^) with increasing amounts of FANCC. Because UNC5A interaction with FANCC occurs via both N-terminal (FANCC^1–306^) and C-terminal (FANCC^307–558^) caspase-mediated cleavage products, we also co-expressed increasing amounts of FANCC fragments with UNC5A [7, 19]. In order to stabilize FANCC, FANCE expression vector was added to each experimental condition [19, 20]. As expected, FANCE expression increased the stability of FANCC, as previously reported [20] but had no effect on UNC5A protein levels (Fig. 3d). Interestingly, neither the co-expression of increasing amounts of FANCC nor its N-terminal fragment, FANCC^1–306^, altered the levels of UNC5A^ICD^ (Fig. 3d, e). In contrast, when cells are transfected with ten times the amount of the C-terminal caspase cleavage product, FANCC^307–558^, UNC5A^ICD^ protein level increased dramatically by almost tenfolds (Fig. 3f). These results suggest that the C-terminal cleavage product of FANCC, FANCC^307–558^, positively impacts UNC5A^ICD^ protein stability. Further investigations are crucial to understand the functional consequence of this increased stability.

### Discussion

In this study, we showed that FANCC and UNC5A are localized together at the tips of neurite-like elongations in cells induced to differentiate. We also observed a requirement for FANCC and UNC5A for cellular differentiation-mediated branching and outgrowth. Given that UNC5 receptors control morphogenesis of neuronal and non-neuronal tissues [21–23], our results suggest that FANCC, via UNC5A, may play a role in branching morphogenesis, or structural organization during organ formation. This idea is supported by the numerous congenital malformations described for patients with FA including those affecting the nervous system [24–26]. Although little is known regarding the role of FA proteins in embryonic development, our previous findings [7] combined to the results presented herein suggest that FANCC might be involved in the UNC5A-mediated apoptotic signal. In fact, FANCC interacts with UNC5A via its C-terminal death domain (DD), which is required for UNC5A-mediated apoptosis in response to Netrin-1 withdrawal [6, 7, 23, 27]. In addition, overexpression of FANCC delays UNC5A-mediated apoptosis, whereas UNC5A levels increase in the presence of FANCC caspase-mediated cleavage products (FANCC^307–558^ or FANCCp47 [19]) [7]. These data suggest that FANCC might be an important regulator of the UNC5A apoptotic signal during tissue morphogenesis. The fact that Unc5A protein levels are reduced in *FancC*^−*/*−^ brains also suggest that FANCC with UNC5A may have critical implications in neuronal tissues. Interestingly, the *FANCC* gene has been associated with entorhinal cortex thickness, a region that is affected early in the progression of Alzheimer’s disease (AD) [28].

Furthermore, significant expression changes in *UNC5A* were found in the posterior cingulate brain region of AD patients, while mutations in *UNC5C* seemed to predispose to late-onset Alzheimer’s disease [29–31]. These data suggest a link between FANCC, the axon-guidance pathway and Alzheimer’s disease thus further highlighting the importance of UNC5A and FANCC in cell death signaling in health and disease conditions.

## Limitations

It is unclear whether interaction between FANCC and UNC5A is required for neurite outgrowth. Further work is needed to determine whether FANCC regulates UNC5A apoptosis during cellular development or axon guidance in vivo.

## Abbreviations

AD: Alzheimer’s disease
FA: Fanconi anemia
FA-A: Fanconi anemia group A
FANCA: Fanconi anemia A
FANCC: Fanconi anemia C
FANCE: Fanconi anemia E
HA: hemagglutinin
UNC5: Uncoordinated-5
UNC5A: Uncoordinated 5A
UNC5C: Uncoordinated 5C
UNC5A^ICD^: Uncoordinated 5A intracellular domain
